# Comparison of Intergrowth-21st and Fenton growth standards to evaluate and predict the postnatal growth in eastern Chinese preterm infants

**DOI:** 10.3389/fped.2023.1259744

**Published:** 2023-11-28

**Authors:** Siyuan Lan, Huanhuan Fu, Chengchen Zhang, Yuyun Chen, Liya Pan, Siqing Song, Yizhi Wang, Li Hong

**Affiliations:** ^1^ Department of Clinical Nutrition, Shanghai Children’s Medical Center, School of Medicine, Shanghai Jiaotong University, Shanghai, China; ^2^ Fujian Children's Hospital (Fujian Branch of Shanghai Children's Medical Center), College of Clinical Medicine for Obstetrics & Gynecology and Pediatrics, Fujian Medical University, Fuzhou, China

**Keywords:** Fenton, Intergrowth-21st, extrauterine growth restriction, intrauterine growth restriction, preterm infants

## Abstract

**Objectives:**

The aim of this article was to compare the differences between Intergrowth-21st (IG-21) and Fenton growth standards in the classification of intrauterine and extrauterine growth restriction (EUGR) in eastern Chinese preterm infants, and detect which one can better relate to neonatal diseases and predict the physical growth outcomes at 3–5 years old.

**Methods:**

Premature infants admitted to a tertiary pediatric hospital in Shanghai, China, from 2016 to 2018 were enrolled. Prenatal information, neonatal diseases during hospitalization, and anthropometric data (weight, height, and head circumference) at birth and at discharge were collected and analyzed. Physical growth outcomes (short stature, thinness, and overweight) were examined by telephone investigations in 2021 at age 3–5 years.

**Results:**

The medium gestational age and birth weight of the included 1,065 preterm newborns were 33.6 weeks and 1,900 g, respectively. The IG-21 curves diagnosed more newborns with small for gestational age (SGA) (19% vs. 14.7%) and fewer newborns with longitudinal EUGR on height (25.5% vs. 27.9%) and head circumference (17.9% vs. 24.7%) compared to Fenton curves. Concordances between Fenton and IG-21 standards were substantial or almost perfect in the classification of SGA and longitudinal EUGR, but minor in cross-sectional EUGR. EUGR identified by Fenton curves was better related to neonatal diseases than IG-21 curves. There were no statistical significances in the prediction of short stature, thinness, and overweight at 3–5 years old between the two charts.

**Conclusions:**

IG-21 growth standards are not superior to Fenton in assessing preterm growth and development in the eastern Chinese population.

## Introduction

The continuous development of perinatal medicine has ensured the survival of a growing number of premature infants, especially those with intrauterine and extrauterine growth restriction (EUGR) (1). The assessment of infant growth is of vital importance since poor growth during infancy can impair both physical and neurodevelopment growth outcomes in later life (2, 3).

Intuitive tools for evaluating and diagnosing the growth of premature infants are growth curves, which can be generally classified into two kinds (4). One is the “standard curve,” namely, the expected growth standard of premature infants, drawn from the growth data of premature infants under the set ideal conditions, such as the WHO growth curves and the Intergrowth-21st (IG-21) growth curves (4). The other is the “reference curve,” which describes the actual growth process of premature infants in a specific period (4). It is usually developed based on the limited retrospective data from decades ago without unified standards, such as the Fenton 2013 and the Olsen 2010 growth charts.

More than 25 growth standards are available for preterm infants (5). Given the absence of internationally recognized local growth charts, Fenton 2013 is the most commonly used growth standard in China. It was generated from retrospective data of nearly 4 million infants in developed countries including America, Australia, Canada, Germany, Italy, and Scotland (6), whereas the IG-21 growth charts, based on a multi-center, multi-ethnic, and prospective study with a sample of infants born to healthy mothers from China, India, Brazil, Oman, Kenya, America, England, and Italy, proposed a supposedly normal growth pattern for preterm infants (7). Recent literature had noticed the overdiagnosis of EUGR by the Fenton 2013 growth charts and considered that growth retardation diagnosed by IG-21 was more associated with neonatal diseases and long-term neurological and physical development delay (2, 8, 9).

Therefore, it is necessary to perform a local validation in the Chinese preterm population to confirm if the IG-21 growth curves are better than Fenton 2013 in these aspects. The objectives of this study were to compare the differences between IG-21 and Fenton growth standards and detect which one can better adapt to eastern Chinese preterm infants.

## Materials and methods

### Study design

Newborns admitted within 24 h after birth from 1 January 2016, to 31 December 2018 to Shanghai Children's Medical Center (SCMC), a national tertiary children's medical center, were eligible to participate in the study. Infants with major congenital malformations or incomplete medical records, dead, or discharged against medical advice were excluded, with a final sample of 1,065 subjects. Follow-up investigations were conducted by telephone inquiries from 1 September 2021 to 31 November 2021, at age 3–5 years. About 548 subjects were considered lost to follow-up due to the absence of contact information, major surgeries during follow-up, inherited metabolic diseases, parental unwillingness, and lack of accuracy in the body measurements. The exclusion of subjects had been explained in the previous study (10). The consent of each parent was obtained. This study was approved by the Ethics Committee of SCMC (SCMCIRB-K2013022).

Gestational age, gender, weight, length, and head circumference (HC) at birth and discharge were collected from the medical charts and converted to Z-scores based on the Fenton 2013 and IG-21 growth standards (6, 7). Weight, height, and HC were all measured by trained nurses using the standard instruments at a designated time. Maternal conditions including hypertensive disorders of pregnancy and gestational diabetes mellitus, neonatal conditions including neonatal asphyxia, neonatal respiratory distress syndrome (NRDS), bronchopulmonary dysplasia (BPD), glycometabolism disorder (both hyperglycemia and hypoglycemia), hyperbilirubinemia, intracranial hemorrhage (ICH), neonatal sepsis, necrotizing enterocolitis (NEC), feeding intolerance, and parenteral nutrition–associated cholestasis (PNAC) were recorded. Height (cm), weight (kg), and body mass index (BMI) at follow-up were gathered through telephone investigations, and Z-scores were calculated using the WHO growth standards.

Small for gestational age (SGA) is defined as birth weight <10th percentile, and large for gestational age (LGA) as birth weight >90th percentile (11). Cross-sectional EUGR is diagnosed as Z-score <−1 standard deviation (SD) at discharge, and longitudinal EUGR as the decline of Z-score >1 SD from birth to discharge (12). At follow-up, height-for-age Z-score <−2 SD is identified as short stature, and BMI-for-age Z-score <−2 SD and BMI-for-age >85th percentile, respectively, as thinness and overweight, according to the WHO growth charts (5).

### Statistical analysis

The categorical variables were described by numbers (percentages). The quantitative variables with a normal distribution were expressed as mean ± SD while those with an abnormal distribution as median (interquartile range). The *χ*^2^ or Fisher's exact test was used for categorical variables, and Mann–Whitney *U*-test was used for abnormally distributed quantitative variables. The Kappa coefficient was used to estimate the agreement between Fenton 2013 and IG-21 growth curves on the classification of intrauterine and extrauterine growth restriction. The Receiver Operating Characteristic (ROC) curves and area under the curves (AUC) were used to evaluate and compare the discriminatory power of Fenton 2013 and IG-21 growth charts in predicting adverse physical growth outcomes at 3–5 years old. Comparisons of ROC curves were conducted by the DeLong test using the medCalc software version 20.0 (MedCalc Software Ltd, Ostend, Belgium). Other analyses were conducted using the SPSS statistical software version 22.0 (SPSS Inc., Chicago, IL, USA). The statistical significance level was set at *P* < 0.05.

## Results

Table 1 presents the perinatal and follow-up information of preterm infants. The medium gestational age and birth weight of the 1,065 preterm infants were 33.6 weeks and 1,900 g, respectively, of which 23.7% were very low birth weight infants (VLBWI, <1,500 g) and 30% were multiple pregnancies. The ranges of gestational age and birth weight were 24^+3^–36^+7 ^weeks, and 665–3,945 g, respectively. The prevalence of neonatal asphyxia, NRDS, BPD, glycometabolism disorder, grade III–IV ICH, neonatal sepsis, NEC, feeding intolerance, and PNAC were 17.2%, 34.6%, 15.9%, 10.2%, 2.3%, 2%, 2.2%, 2.3%, and 3.1%, respectively. The medium age at discharge and follow-up were 35.9 weeks and 4.25 years. The average Z-score for weight was −0.03 ± 1.11 SD and the medium Z-score for height was 0.12 SD at follow-up. Of the 527 subjects with complete follow-up data, 3.8%, 6.6%, and 15% were identified as short stature, thinness, and overweight, respectively.

**Table 1:** The perinatal and follow-up information of preterm infants.

Perinatal characteristics (*n* = 1065)	Value
Gestation age (w)	33.6 (31.8, 34.7)
Birth weight (g)	1900 (1535, 2175)
Length at birth (cm)	40 (43, 45)
HC at birth (cm)	28 (30, 31.5)
Male sex (%)	583 (54.7)
Birth weight <1500 g (%)	252 (23.7)
Multiple births (%)	320 (30)
Hypertensive disorders of pregnancy (%)	262 (24.6)
Gestational diabetes mellitus (%)	151 (14.2)
Neonatal asphyxia (%)	183 (17.2)
NRDS (%)	368 (34.6)
BPD (%)	169 (15.9)
Glycometabolism disorder (%)	109 (10.2)
Hyperbilirubinemia (%)	886 (83.2)
Grade III–IV of ICH (%)	25 (2.3)
Neonatal sepsis (%)	21 (2)
NEC (%)	23 (2.2)
Feeding intolerance (%)	25 (2.3)
PNAC (%)	33 (3.1)
Corrected gestational age at discharge (w)	35.9 (36.7, 37.9)
Weight at discharge (g)	2170 (2030, 2390)
Length at discharge (cm)	44 (46, 47.7)
HC at discharge (cm)	31 (32, 33)
Follow-up characteristics (*n* = 527)	Value
Age at follow-up (y)	4.25 (3.41, 5.12)
Weight Z-score at follow-up (SD)	−0.03 ± 1.11
Height Z-score at follow-up (SD)	0.12 (−0.61, 0.89)
Body Mass Index Z-score at follow-up (SD)	−0.27 (−0.97, 0.46)
Short stature at follow-up (%)	20 (3.8)
Thinness at follow-up (%)	35 (6.6)
Overweight at follow-up (%)	79 (15)

HC, head circumference; NRDS, neonatal respiratory distress syndrome; BPD, bronchopulmonary dysplasia; ICH, intracranial hemorrhage; NEC, necrotizing enterocolitis; PNAC, parentarel nutrition associated cholestasis.

**Figure 1:**
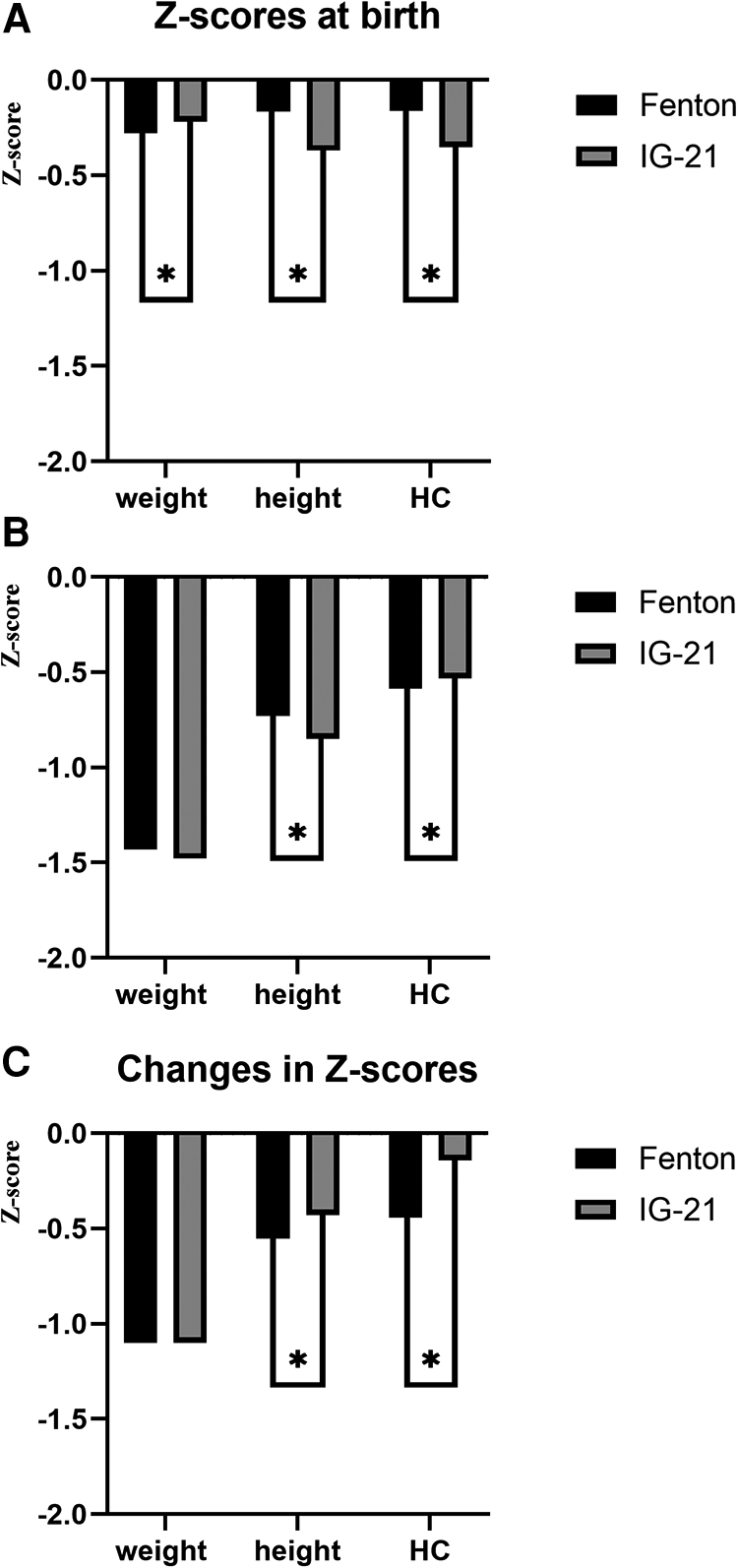
(**A**) Z-scores at birth of preterm infants using Fenton and IG-21 growth curves. (**B**) Z-scores at discharge of preterm infants using Fenton and IG-21 growth curves. (**C**) Changes in Z-scores of preterm infants during hospitalization using Fenton and IG-21 growth curves.

The classification of SGA, LGA, and EUGR in preterm infants by Fenton or IG-21 growth charts is shown in Table 2. The agreements of distribution of SGA and LGA by these two charts were almost perfect, although the incidences of SGA were significantly higher when using IG-21 growth curves. Slight concordances were noticed in the diagnosis of cross-sectional EUGR, while substantial concordances were observed in the classification of longitudinal EUGR. The incidences were remarkably higher in cross-sectional EUGR for height, and lower in longitudinal EUGR for height and HC when estimated by IG-21 curves than Fenton growth standards. As the gestational age increased, the consistencies of SGA and longitudinal EUGR for weight increased, while the consistencies of longitudinal EUGR for height decreased. Z-scores for weight were significantly lower, and Z-scores for height and HC were significantly higher at birth when Fenton curves were used (Figure 1A). At discharge, the IG-21 curves identified significantly lower Z-scores for height and higher Z-scores for HC (Figure 1B). IG-21 showed less decrease of Z-score in height and HC than Fenton growth standards (Figure 1C).

**Table 2:** The classification of SGA, LGA, and EUGR in preterm infants by Fenton or IG-21 growth charts.

Gestational age		Fenton	IG-21	*P*-value	Kappa
<32 weeks (*n *= 276)	SGA	16 (5.8)	31 (11.2)	<0.001	0.654
	LGA	14 (5.1)	14 (5.1)	1.000	0.850
	Cross-sectional EUGR for weight	191 (69.2)	196 (71)	0.709	0.006
	Cross-sectional EUGR for height	124 (44.9)	119 (43.1)	0.727	0.037
	Cross-sectional EUGR for HC	115 (41.7)	86 (31.2)	0.013	0.018
	Longitudinal EUGR for weight	209 (75.7)	204 (73.9)	0.500	0.664
	Longitudinal EUGR for height	126 (45.7)	112 (40.6)	0.007	0.823
	Longitudinal EUGR for HC	109 (39.5)	91 (33)	0.001	0.797
32–34 weeks (*n *= 343)	SGA	46 (13.4)	60 (17.5)	0.003	0.778
	LGA	10 (2.9)	10 (2.9)	1.000	1.000
	Cross-sectional EUGR for weight	244 (71.1)	247 (72)	0.864	0.019
	Cross-sectional EUGR for height	142 (41.4)	172 (50.1)	0.025	0.021
	Cross-sectional EUGR for HC	114 (33.2)	123 (35.9)	0.529	−0.037
	Longitudinal EUGR for weight	224 (65.3)	227 (66.2)	0.749	0.748
	Longitudinal EUGR for height	94 (27.4)	91 (26.5)	0.711	0.785
	Longitudinal EUGR for HC	89 (25.9)	51 (14.9)	<0.001	0.612
≥34 weeks (*n *= 446)	SGA	95 (21.3)	111 (24.9)	<0.001	0.874
	LGA	16 (3.6)	17 (3.8)	1.000	0.906
	Cross-sectional EUGR for weight	297 (66.6)	295 (66.1)	0.942	0.006
	Cross-sectional EUGR for height	161 (36.1)	204 (45.7)	0.003	0.049
	Cross-sectional EUGR for HC	136 (30.5)	143 (32.1)	0.656	0.056
	Longitudinal EUGR for weight	202 (45.3)	186 (41.7)	0.033	0.772
	Longitudinal EUGR for height	77 (17.3)	69 (15.5)	0.215	0.738
	Longitudinal EUGR for HC	65 (14.6)	49 (11)	0.002	0.759
Total (*n *= 1,065)	SGA	157 (14.7)	202 (19)	<0.001	0.816
	LGA	40 (3.8)	41 (3.8)	1.000	0.910
	Cross-sectional EUGR for weight	723 (67.9)	738 (69.3)	0.811	0.038
	Cross-sectional EUGR for height	427 (40.1)	495 (46.5)	0.003	0.036
	Cross-sectional EUGR for HC	365 (34.3)	352 (33.1)	0.580	0.140
	Longitudinal EUGR for weight	635 (59.6)	617 (57.9)	0.127	0.760
	Longitudinal EUGR for height	297 (27.9)	272 (25.5)	0.009	0.796
	Longitudinal EUGR for HC	263 (24.7)	191 (17.9)	<0.001	0.739

The association between neonatal diseases and the diagnosis of SGA and EUGR by Fenton and IG-21 growth curves is presented in Table 3. There were significant differences in morbidity of NRDS, glycometabolism disorder, and feeding intolerance between SGA and non-SGA divided by both Fenton and IG-21 growth charts. PNAC was only related to SGA diagnosed by IG-21 growth charts. BPD, feeding intolerance, and PNAC were associated with cross-sectional EUGR defined by Fenton rather than IG-21 growth charts. In addition, neonatal asphyxia, glycometabolism disorder, and grade III–IV ICH were related to Fenton but not IG-21 longitudinal EUGR.

**Table 3:** Association between neonatal diseases and the diagnosis of SGA and EUGR by Fenton and IG-21 growth curves.

Neonatal diseases	SGA		Cross-sectional EUGR on weight		Longitudinal EUGR on weight	
	Fenton (*n* = 207)	IG-21 (*n* = 202)	Fenton (*n* = 732)	IG-21 (*n* = 738)	Fenton (*n* = 635)	IG-21 (*n* = 617)
Neonatal asphyxia (%)	21 (13.4)	31 (15.3)	135 (18.4)	122 (16.5)	121 (19.1)^c^	112 (18.2)
NRDS (%)	34 (21.7)^a^	55 (27.2)^a^	250 (34.2)	255 (34.6)	281 (44.3)^c^	266 (43.1)^c^
BPD (%)	21 (13.4)	33 (16.3)	140 (19.1)^b^	124 (16.8)	149 (23.5)^c^	132 (21.4)^c^
Glycometabolism disorder (%)	31 (19.7)^a^	32 (15.8)^a^	82 (11.2)	75 (10.2)	82 (12.9)^c^	72 (11.7)
Hyperbilirubinemia (%)	129 (82.2)	167 (82.7)	611 (83.5)	621 (84.1)	562 (88.5)^c^	545 (88.3)^c^
Grade III–IV of ICH (%)	4 (2.5)	4 (2)	18 (2.5)	15 (2)	21 (3.3)^c^	19 (3.1)
Neonatal sepsis (%)	4 (2.5)	6 (3)	17 (2.3)	11 (1.5)	14 (2.2)	13 (2.1)
NEC (%)	3 (1.9)	4 (2)	20 (2.7)	16 (2.2)	20 (3.1)^c^	19 (3.1)^c^
Feeding intolerance (%)	73 (46.5)^a^	101 (50)^a^	314 (42.9)^b^	271 (36.7)	266 (41.9)^c^	241 (39.1)^c^
PNAC (%)	7 (4.5)	12 (5.9)^a^	33 (4.5)^b^	24 (3.3)	32 (5)^c^	25 (4.1)^c^

^a^A significant difference between SGA and non-SGA divided by Fenton or IG-21 curves.

^b^A significant difference between cross-sectional EUGR amd non-EUGR on weight divided by Fenton or IG-21 curves.

^c^A significant difference between longitudinal EUGR and non-EUGR on weight divided by Fenton or IG-21 curves.

SGA, small for gestational age; EUGR, extrauterine growth restriction; NRDS, neonatal respiratory distress syndrome; BPD, bronchopulmonary dysplasia; ICH, intracranial hemorrhage; NEC,necrotizing enterocolitis; PNAC, parentarel nutrition associated cholestasis.

**Figure 2:**
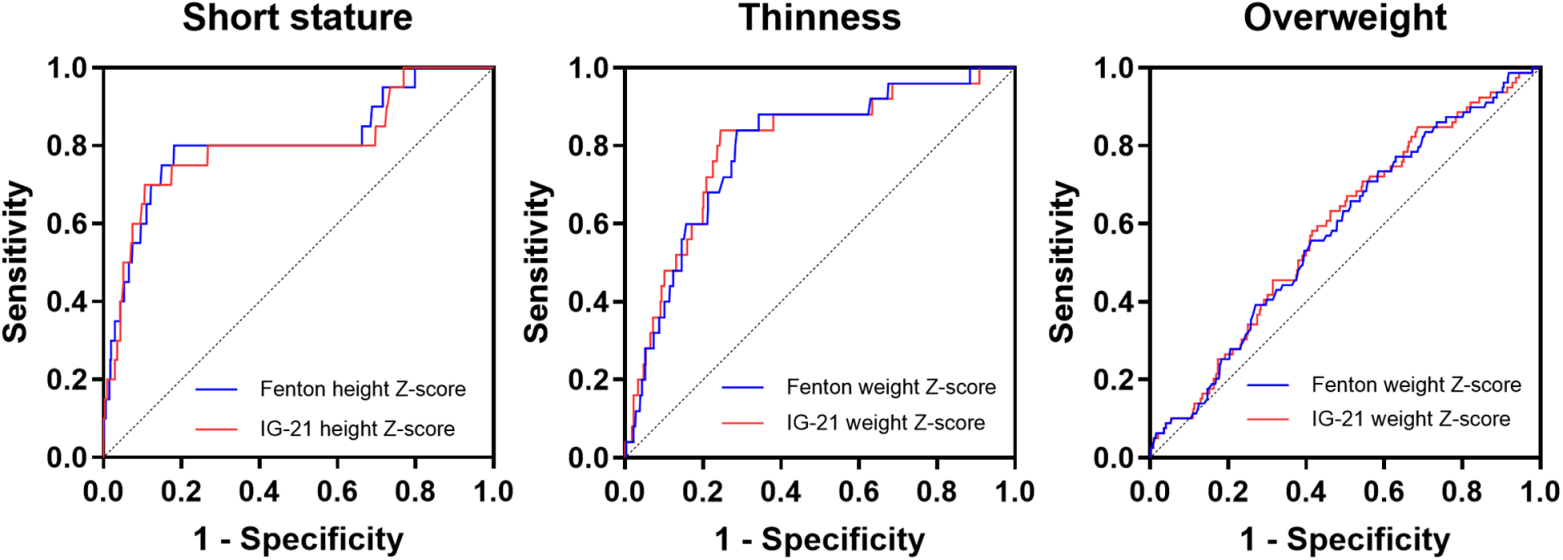
ROC curves of the prediction of short stature, thinness, and overweight at 3–5 years old according to birth height Z-scores using Fenton or IG-21 growth charts.

Table 4 and Figure 2 show the prediction of thinness, short stature, and overweight at 3–5 years old according to birth weight or height Z-scores using Fenton or IG-21 growth charts. Weight Z-scores at birth were identified as risk factors for thinness and overweight, and height Z-scores at birth as risk factors for short stature. The AUCs of weight and height Z-scores at birth based on Fenton curves were significantly larger than those based on IG-21 curves (Figure 2). However, the DeLong test showed no statistical discrepancies between the ROCs of Fenton and IG-21 curves in the prediction of thinness, short stature, and overweight at 3–5 years old.

**Table 4:** Prediction of thinness, short stature, and overweight at 3–5 years old according to birth weight or height Z-scores using Fenton or IG-21 growth charts.

	B	*P*-value	Exp (B)	95% CI	AUC
Short stature					
Fenton birth height Z-score	−1.093	<0.001	0.335	(0.222–0.505)	0.808
IG-21 birth height Z-score	−0.606	<0.001	0.545	(0.405–0.734)	0.801
Thinness					
Fenton birth weight Z-score	−1.09	<0.001	0.336	(0.219–0.516)	0.800
IG-21 birth weight Z-score	−0.785	<0.001	0.456	(0.321–0.647)	0.791
Overweight					
Fenton birth weight Z-score	0.347	0.007	1.414	(1.098–1.822)	0.588
IG-21 birth weight Z-score	0.28	0.024	1.323	(1.037–1.686)	0.581

CI, confidence interval.

## Discussion

Recently, a retrospective cohort study discovered that the postnatal growth of preterm infants in Shandong province (northern China) was considerably higher than that of the IG-21 growth standards at 40–64 weeks (13). Zhang et al. (14) also investigated 24,375 infants from 13 cities in China and noticed an inconsistency between the updated Chinese birth size charts and the IG-21 references. However, the domestic data by now are still insufficient to determine whether the IG-21 curves can replace Fenton as an evaluation instrument for premature infants in China. In this regard, this article provides information for addressing this issue by comparing the applications of Fenton and IG-21 curves in eastern Chinese preterm infants.

To our best knowledge, many studies abroad had explored the application of IG-21 curves in different populations. The studies by Anne et al. (9), Reddy et al. (15), and Estañ-Capell et al. (16) provided the data of the application of IG-21 growth charts in early preterm birth (<32 weeks); González García et al. (17, 18) and Yazici et al. (19) focused on very low birth weight infants (<1,500 g). Most research studies were limited to specific high-risk populations and overlooked the middle and late preterm infants, which occupied a large majority of preterm infants. In this article, preterm infants with a wide range of gestational age from 24^+3^ to 36^+7 ^weeks and birth weight from 665 to 3,945 g were included.

The divisions of SGA and LGA based on IG-21 or Fenton charts in this research were similar to some previous studies (9, 20, 21). The incidences of SGA assessed by IG-21 curves were significantly higher than that assessed by Fenton, which indicated that the IG-21 curves classified more preterm infants as SGA, in the case of high consistency between the two curves, especially in early preterm newborns. The higher birth standard of IG-21 curves can be attributed to the strict inclusion criteria in their data, which were infants born to healthy mothers without congenital malformations, severe maternal obesity or morbidity, maternal smoking, or ultrasound evidence of fetal growth restriction (7). Early preterm newborns born to healthy mothers might be inherently a false proposition since extremely premature births usually go hand in hand with unhealthy factors. Consequently, only 408 eligible neonates were enrolled in the supplement data of IG-21 very preterm growth references (22). Meanwhile, SGA divided by Fenton or IG-21 curves was related to approximately the same neonatal diseases, which means that the IG-21 curves may not accurately discriminate critical or unhealthy preterm infants better than Fenton curves.

The study by El Rafei et al. showed that very preterm newborns with discharge weight <10th percentile using Fenton charts were more than that using IG-21 curves (23). However, there were no significant differences in the incidences of cross-sectional EUGR by Fenton and IG-21 curves in this study, and concordances between the two curves were quite low. Neonatal diseases were more closely related to cross-sectional weight EUGR by Fenton instead of IG-21 curves, indicating that the diagnosis of EUGR by IG-21 growth chart was invalid in a sense. As same as the study conducted by Kim etc. (21), the incidences of longitudinal EUGR in early, middle, and late preterm infants identified by Fenton were higher than that by IG-21 curves, and the consistencies between the two curves were relatively high, which means that the Fenton charts classified more preterm infants as longitudinal EUGR. Both were associated with several neonatal diseases, but Fenton, rather than the IG-21 longitudinal EUGR, was affected by neonatal asphyxia and grade III–IV ICH.

The prediction of prognosis using Fenton or IG-21 curves had already been assessed in many previous studies (1, 5, 18, 24). Yitayew et al. (1). and Cordova et al. (24) compared the relation between growth failure identified by IG-21 or Fenton and neurodevelopmental delay at 12, 18, and 24 months and reached different conclusions. Lebrão et al. (5) indicated that the IG-21 charts were slightly better than Fenton to predict adverse physical growth outcomes at 12 months, while González García et al. (18) found no statistical differences between the two charts in predicting the body build of VLBWI at 2 years. However, the long-term prognosis after 2 years was barely discussed. In this case, this study followed the physical development results of children aged 3–5 years old, when the vast majority of catch-up growth had already been completed (25). The results showed that for the prediction of obesity, short stature, and thinness at age 3–5 years, the Fenton curves had slightly higher AUCs than the IG-21 curves, yet no statistical differences were observed. Since obesity usually reflects the recent nutritional status and is easier to be interfered with some acquired factors, such as dietary habits and exercise, the AUCs of obesity were less than 0.6.

Although most studies abroad had shown that the IG-21 curves outperformed Fenton in some cases, this study did not draw similar conclusions, probably because of the study population and nutrition policies.

China, as the largest developing country in the world, has a large diversity in regional demographic characteristics and healthcare levels (26). This study was conducted in Shanghai, eastern China, while the Chinese data contained in the IG-21 curves were selected from Beijing, the capital located in northern China. Wu et al. (26) pointed out that the physique of the northern population was generally larger than average, and local growth curves may be the best way to evaluate local newborns. This study demonstrated that the IG-21 growth standards may be not perfect for eastern Chinese preterm infants, but it could not represent the other districts of the country.

On the other hand, the data of Fenton curves were derived from developed countries, and Shanghai is one of the most developed cities in China, which may explain the closer relation to neonatal diseases when using Fenton curves compared with IG-21 curves. In addition, SCMC is a first class specialist children's tertiary hospital with advanced medical technologies and healthcare levels, receiving critically ill premature infants from eastern China including Shanghai, Zhejiang, Jiangsu province, etc. VLBWI and early preterm infants occupied 23.7% and 25.9% of the study population, respectively. The IG-21 curves were short of data on small gestational age newborns compared to Fenton charts, which may also contribute to the results of this study.

The nutritional policies for premature infants were updated synchronously with the Chinese guidelines for nutrition support in neonates (27, 28). Infants enrolled in this research were born between 2016 and 2018, during which nutritional assessments were conducted using the Fenton 2013 curves. Premature infants identified with growth retardation by Fenton charts during hospitalization may be implemented with more proactive nutritional therapies, which may have an impact on the prognosis of these children.

This is a single-center retrospective study with a dropout rate of 50.5% during follow-up telephone investigations. However, this is the first article comparing Fenton and IG-21 curves in the eastern Chinese preterm population. A large number of infants with a wide range of gestational age and birth weight were included and followed up until 3–5 years old, which can serve as a reference for clinical practice.

## Conclusions

Concordances between the IG-21 and the Fenton growth standards in the classification of SGA, LGA, and longitudinal EUGR were high. IG-21 curves are not superior to Fenton in the assessment of preterm growth and the prediction of physical development outcomes at 3–5 years old in the eastern Chinese population.

## Data Availability

The raw data supporting the conclusions of this article will be made available by the authors, without undue reservation.
